# Dental Versus Skeletal Methods for Age Estimation in Growing Individuals: A Systematic Review

**DOI:** 10.7759/cureus.98020

**Published:** 2025-11-28

**Authors:** Azhar A Khan, Sumit Bhateja

**Affiliations:** 1 Oral Pathology and Microbiology, Manav Rachna Dental College, Manav Rachna International Institute of Research and Studies, Faridabad, IND; 2 Oral Medicine and Radiology, School of Dental Sciences, Manav Rachna International Institute of Research and Studies, Faridabad, IND

**Keywords:** age estimation, chronological age, dental age, forensic odontology, skeletal age

## Abstract

Accurate age estimation is crucial in forensic, clinical, and legal contexts. Dental age (DA) and skeletal age (SA) are commonly used markers, yet their relative accuracy across populations remains unclear. This systematic review aimed to compare the accuracy of DA and SA estimation methods relative to chronological age (CA) in children and adolescents. A comprehensive search of PubMed, Scopus, EBSCOhost, and the Cochrane Library identified studies evaluating dental methods such as Demirjian, Willems, Cameriere, and Nolla, as well as skeletal methods including Fishman, Greulich and Pyle, cervical vertebral maturation (CVM), and Gilsanz-Ratib. Studies involving individuals aged 6-19 years were analyzed, and the risk of bias was assessed using the modified Newcastle-Ottawa Scale. Nine studies from diverse populations, including Turkey, Egypt, and South Africa, were included. The findings revealed that DA methods, particularly Willems and Nolla, demonstrated a higher correlation with CA than SA methods, which often underestimated age in older children. Accuracy varied according to age, sex, and population, while combined approaches improved reliability. Overall, DA estimation methods generally outperform SA techniques in early to mid-adolescents, though population-specific calibration and combined DA+SA approaches enhance accuracy. Careful method selection remains essential in forensic and clinical applications.

## Introduction and background

Accurate age estimation in children and adults is a cornerstone of forensic odontology, orthodontics, pediatric dentistry, civil law, and identification of individuals with disputed or unknown birth records [1,2]. Chronological age (CA) is the age computed from the date of birth and is the ideal benchmark. However, biological maturation often lags or leads CA owing to genetic, nutritional, environmental, and health‑related factors, making reliance on CA alone problematic for many legal or clinical applications [3].

Two principal biological markers are commonly used to estimate developmental age: dental age (DA), based on tooth formation or eruption, and skeletal age (SA), based on maturation of bones. Dental development is frequently assessed using methods such as Demirjian, Willems, Cameriere (open‑apex), or Nolla, each employing different staging or measurement systems based on panoramic radiographs. For example, several studies have shown that the Demirjian method tends to overestimate CA in many populations, whereas the Willems method may perform more closely, with lower mean error [4-7].

SA estimation methods include assessment of hand‑wrist bones (e.g., Fishman's method, Greulich and Pyle atlas) and cervical vertebral maturation (CVM) on lateral cephalograms. These skeletal markers reflect ossification stages and fusion of growth plates, which are less influenced by dental pathology but can be affected by overall growth rate, hormonal changes, and pubertal timing. Studies in Yemeni children, Lebanese populations, and others demonstrate a high correlation of SA with CA but often show under- or over-estimation depending on age group and sex [8].

Comparative studies that assess both DA and SA within the same cohort allow evaluation of which method aligns more closely with CA. For example, a study of 100 Indian children (9-14 years old) found that both Demirjian's and Willems' dental methods had high accuracy when compared with CA, whereas Fishman's skeletal method overestimated CA in both sexes. Another study in Finland and Turkey comparing Cameriere's open‑apex method with Demirjian's method found that the latter often overestimated age, whereas Cameriere underestimated age in some age groups. Studies in Germany likewise report that Demirjian's method may be superior to Cameriere's in that particular population [9-11]. Nevertheless, no single method has shown universal superiority: accuracy depends heavily on population, sex, age interval, imaging method, and observer calibration. Errors in estimation, i.e., either over‑ or underestimations, may carry practical consequences: in legal contexts (e.g., if a threshold age determines criminal responsibility), in orthodontic treatment timing, and when assessing growth disorders. Thus, systematic evaluation of DA vs. SA methods in diverse populations is essential [12-15].

The current review aims to synthesize recent evidence on the accuracy of DA and SA estimation methods in children, focusing on their accuracy relative to CA. Specifically, this review will assess which methods tend to provide estimates closer to CA across age groups (especially early to mid adolescence), determine how sex and ethnicity affect estimation error, and consider the relative strengths and limitations of DA vs. SA approaches. The goal is to inform forensic, clinical, and legal practice towards selecting the most reliable age estimation method in a given context [16-21].

## Review

Materials and methods

Protocol Registration

**Figure 1:**
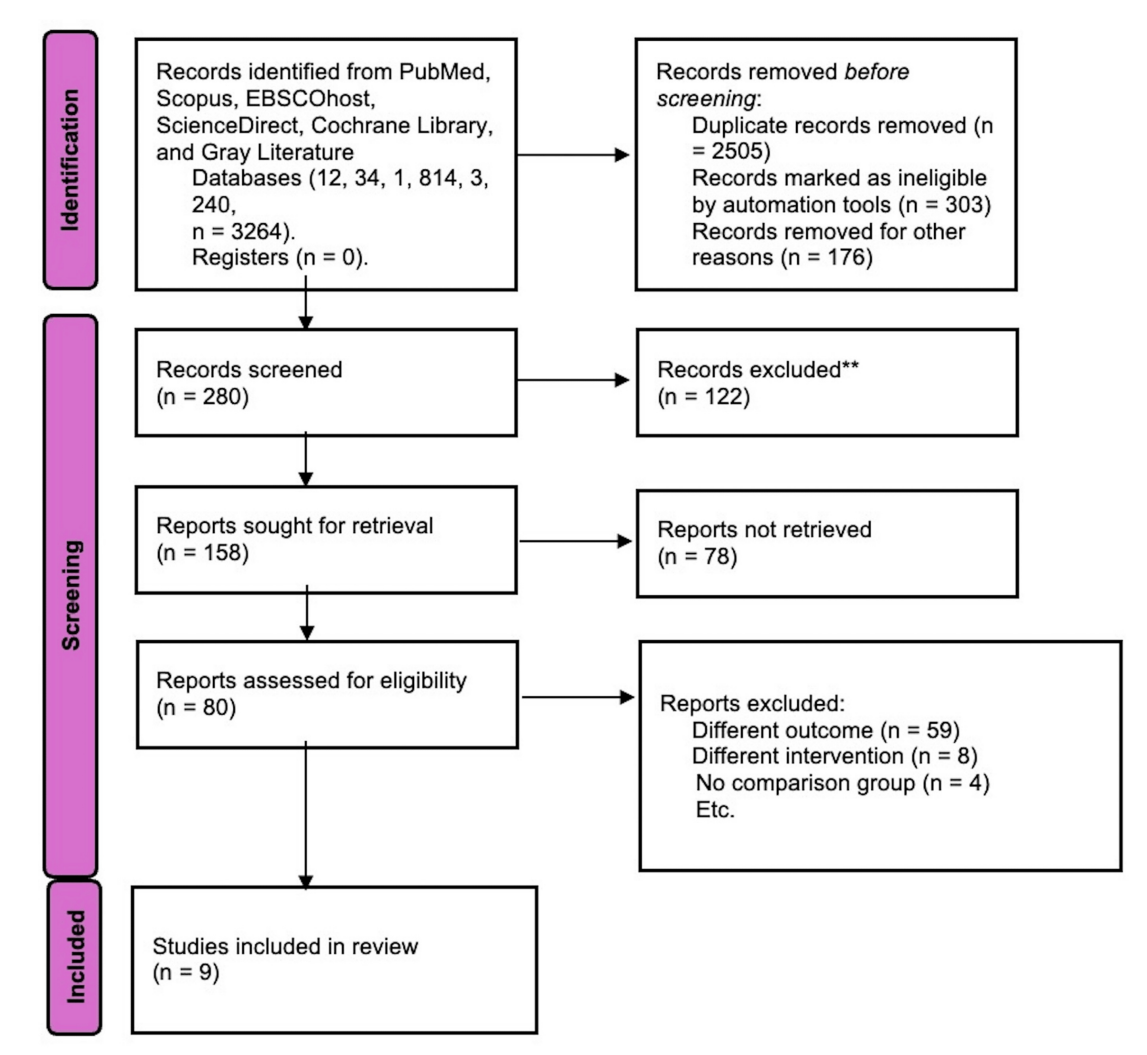
PRISMA 2020 flow diagram, showing the identification, screening, eligibility, and inclusion of studies as per PRISMA 2020 guidelines. n: number of studies; PRISMA: Preferred Reporting Items for Systematic Reviews and Meta-Analyses [22].

A systematic review was conducted using the PICOS (Population, Intervention, Comparison, Outcomes, and Study) framework. A comprehensive literature search found no prior reviews on this topic. The study followed PRISMA (Preferred Reporting Items for Systematic Reviews and Meta-Analyses) 2020 guidelines (Figure 1) and was pre-registered with the Open Science Framework. Methodology was based on the Cochrane Handbook. Before framing the research question, the authors evaluated the evidence gap and the forensic relevance of the topic [10].

Focused Question

Using the PICOS framework and the Cochrane Handbook for Systematic Reviews of Interventions as a guide, this review question was generated [19,20]. By mutual consent, a group of authors, AK and SB, designed the research question, taking into account the availability, significance, and need for systematic reviews of relevant themes. As a result, this review compares the evaluation of the accuracy of various DA and SA estimation methods in young adults and includes in vitro research. Thus, the research question is, "Is there a correlation in terms of accuracy between dental and skeletal age?"

Literature Search Strategies

Scopus, PubMed, EBSCOhost, and the Cochrane Library were included as major search engines for article retrieval. The search strategy comprised text words and MeSH terms, which consisted of the following keywords: "dental age", "skeletal age", and "estimation methods". The following search terms were used to search for articles: (Dental age) AND (skeletal age) AND (Adult population OR "Young population"), using the All Fields. Articles were initially filtered according to the title and abstract, and papers that matched the inclusion criteria were considered relevant.

Inclusion criteria include children and adolescents aged 6-19 years and focused on studies that utilized dental methods such as Cameriere, Demirjian, Willems, and Nolla, and/or skeletal methods including Fishman, Greulich and Pyle, Gilsanz-Ratib, and CVM. Eligible studies were those that reported the accuracy or correlation of these methods with CA. Only original cross-sectional or retrospective studies published in English were included.

Exclusion criteria include studies involving participants over 19 years old or mixed-age groups without separate pediatric data. Non-radiographic methods, reviews, case reports, abstracts, editorials, and studies lacking sufficient data or an English full text were also excluded.

Quality Assessment of the Included Studies

Two assessors, AK and SB, independently assessed the risk of bias for each included study using the Newcastle-Ottawa scale, as per the PRISMA 2020 statement.

Data Collection/Extraction Process

AK and SB extracted the data independently, concentrating on the types of studies and related factors; they then reviewed and finalized the data selection and resolved disagreements through discussion. Data relevant to the inclusion criteria were retrieved, and studies were excluded if data disaggregation was not possible. Data accuracy was confirmed by all two authors.

Results

After the initial database search, 3,264 studies were identified. Following an examination of titles and abstracts, 3,255 studies were excluded. After duplicates were removed, both reviewers excluded studies and reviews that did not meet the eligibility criteria, as shown in Table 1. Any uncertainties were resolved through discussion between the reviewers. The remaining nine studies [12-20] were included in this systematic review, focusing on the comparative accuracy of age estimation by skeletal method and DA estimation methods. These studies specifically compared the SA estimation method with other DA estimation techniques in young adults to assess their relative effectiveness.

**Table 1:** Outcomes of the studies included in the systematic review. OPG: orthopantomogram; CVM: cervical vertebral maturation; G&P: Greulich and Pyle atlas; GRA: Gilsanz–Ratib atlas; SA: skeletal age; DA: dental age.

Author (Year, Country)	Population	Dental Method	Skeletal Method	Age Group	Main Findings/Conclusion
Güler et al. [12], 2023, Turkey	216 radiographs (130 females, 86 males)	Cameriere (panoramic, open-apex)	Cervical vertebral maturation (CVM, 4th cervical vertebra)	9–14.99 yrs	SA is more accurate than DA in children aged 9–12.99 years
Bhadana et al. [13], 2019, India	100 children	Demirjian & Willems (OPG)	Fishman (hand-wrist)	9–14 yrs	DA is more accurate than SA
Elmaguid Kaka et al. [14], 2025, Egypt	140 children (70 girls, 70 boys)	Willems, Cameriere (OPG)	Greulich & Pyle atlas (hand-wrist)	8–16 yrs	Willems + G&P is the most accurate; combination is recommended
Magat & Ozcan [15], 2022, Turkey	284 patients	Dental age (panoramic)	CVM and hand-wrist	9–19 yrs	DA is more accurate than SA
Saraç et al. [16], 2024, Turkey	Children	Cameriere European method	Gilsanz-Ratib atlas (hand-wrist)	9–15 yrs	Cameriere is more accurate in younger ages; GRA is better in older ages
Lashin et al. [17], 2025, Egypt	176 children	Nolla (OPG)	Fishman (hand-wrist)	8–16 yrs	Nolla more accurate than Fishman
Ghergie et al. [18], 2025, Romania	73 patients (Class II malocclusion)	Demirjian + chronology of eruption	Baccetti CVM (lateral cephalogram)	Children/adolescents	CVM most accurate
Galic et al. [19], South Africa, 2025	556 children (Black and White)	Cameriere (open-apex, tooth heights)	Carpal bones, ulna, radius (hand-wrist)	6–16 yrs	Cameriere more accurate
Bravo et al. [20], Turkey, 2023 (Forensic Sci Med Pathol)	Turkish population	Cameriere (OPG)	Carpal bones, ulna, radius	Not specified	Cameriere more accurate

Characteristics of the Individual Studies

A study conducted by Güler et al. [12] aimed to compare Cameriere's DA and SA methods in estimating CA in children. A total of 216 radiographs (130 females, 86 males; ages 9.00-14.99 years) from northwestern Turkey were analyzed. DA was assessed using Cameriere's open-apex method on panoramic radiographs, while SA was determined via the fourth cervical vertebra on lateral cephalograms. The mean CA was 12.96±0.30, DA was 12.74±0.68, and SA was 12.89±0.89. DA tended to overestimate age in younger children and underestimate it in older age groups, with variations by sex. SA showed significant underestimation in older children. The findings suggest that SA may offer more accurate age estimations than DA in children aged 9.00-12.99 years [12].

A study by Bhadana et al. [13] aimed to compare CA with DA using Demirjian's and Willem's methods and SA using Fishman's method in 100 children aged 9-14 years. CA was determined from birth certificates, DA from orthopantomograms, and SA from left hand-wrist radiographs. The mean CA was 12.37±1.34 years, while DA was nearly equal for both dental methods. SA, however, showed a higher mean of 13.03 ± 1.34 years. Willem's method demonstrated the highest correlation with CA (r = 0.885), while Fishman's method showed the lowest (r = 0.728), indicating that dental methods were more accurate than skeletal in this population [13].

A study conducted by Elmaguid Kaka et al. (2025) [14] assessed the accuracy of the Willems, Cameriere, and Greulich and Pyle methods for age estimation in 140 Egyptian children (70 boys, 70 girls) aged 8-16 years. The Willems method slightly underestimated CA in both sexes, with no significant difference between the sexes. Cameriere's method significantly underestimated CA in both boys and girls. The Greulich and Pyle atlas slightly overestimated age in boys and underestimated it in girls, with no statistical significance. The Willems method showed the lowest mean absolute error (<1 year), indicating higher accuracy. Combining the Willems and Greulich and Pyle methods may enhance age estimation reliability in the Egyptian pediatric population [14].

Magat and Ozcan (2022) [15] evaluated 284 Turkish individuals aged 9-19 years to assess CA using dental maturation (DM), CVM, and hand-wrist maturation (HWM). Panoramic, lateral cephalometric, and hand-wrist radiographs were analyzed. Significant correlations were observed between CA and all three indicators (P < 0.05). DM showed no sex-related differences, whereas CVM and HWM varied by sex. DM demonstrated the highest accuracy, with estimates closely matching CA, indicating its reliability as a practical tool for medical, orthodontic, and forensic age estimation [16].

A study by Saraç et al. (2024) [16] compared different methods to estimate age in children aged 9-15 years. Researchers used the Gilsanz-Ratib atlas (GRA) for bone age, Cameriere's European method for DA, and measured parts of the lower jawbone. The results showed that Cameriere's method worked better for younger children, while the GRA was more accurate for older ones. Two jaw measurements, condylar height and tangential ramus height, had a strong link with age. These findings suggest that jawbone measurements can be helpful tools in forensic science to support age estimation [16].

Lashin et al. (2025) [17] assessed age estimation in 176 Egyptian children (8-16 years) using dental mineralization (Nolla method) and skeletal maturation (Fishman method). The Nolla method exhibited a stronger correlation with CA, slightly underestimating it by -0.21 years, while the Fishman method slightly overestimated it by 0.17 years. Higher intraclass correlation coefficients and superior model fit indicated that Nolla's dental assessment more accurately reflects CA in this population [18].

Ghergie et al. [18] investigated the correlation between CA, DA, and CVM in 73 Class II malocclusion patients (31 males, 42 females). Orthopantomograms and lateral cephalograms were used, with DA assessed via Demirjian and Chronology of Eruption methods and SA via Baccetti's CVM staging. Significant correlations between CVM and both CA and DA indicate that DA can reliably supplement skeletal assessment for growth evaluation and orthodontic treatment planning [18].

A study by Galic et al. (2024) [19] compared the accuracy of original and newly developed South African formulas for age estimation in Black South African (BSA) and White South African (WSA) children and adolescents. The research focused on dental and hand-wrist radiographs, assessing open apices, tooth heights, and the area of carpal bones. A sample of 556 subjects, aged 6-15 years, was analyzed. The findings revealed that the combined dental and hand-wrist formula provided the best performance for age estimation, particularly for WSA males. However, the new South African formulas showed promising improvements in accuracy, especially for males, suggesting their potential suitability for age estimation in South African populations [19].

Bravo Molina (2022) [20] developed a formula to estimate the CA of Turkish children by measuring developing teeth, carpal bones, and the epiphyses of the ulna and radius. The number of closed-apex teeth and the distance between open apices were measured using ImageJ, while carpal bone areas were assessed with X-rays. The total carpal bone area (Bo) and the carpal area (Ca) were calculated, and their ratio was used for normalization. A new regression model was developed, which showed 72.80% accuracy in predicting CA, outperforming existing formulas and demonstrating effectiveness in Turkish children's age estimation [20].

Assessment of Risk of Bias

Using the modified Newcastle-Ottawa Scale as shown in Table 2, most included studies demonstrated a moderate risk of bias, with only a few rated low. Populations were typically well defined with clear age ranges, strengthening selection validity [22]. However, many studies failed to account for confounding factors such as sex, ethnicity, and nutrition, thereby limiting comparability. While most applied age estimation methods are consistently against CA, stronger studies from Egypt and Eastern Anatolia used larger, balanced samples and multiple methods, enhancing reliability compared to smaller or retrospective designs [23].

**Table 2:** Modified Newcastle–Ottawa Scale (NOS) risk of bias assessment. Risk of bias assessment of the included studies using the Modified Newcastle–Ottawa Scale (NOS). Scores: Selection (0–4), Comparability (0–2), Outcome/Exposure (0–3), Total (0–9). Risk categories: low (7–9), moderate (4–6), high (0–3) [21,22].

Study	Selection (0–4)	Comparability (0–2)	Outcome/Exposure (0–3)	Total (0–9)	Risk of Bias
Güler et al. 2023 (Turkey) [12]	4	1	2	7	Moderate
Bhadana et al. 2019 (India) [13]	3	1	2	6	Moderate
Elmaguid Kaka et al. 2025 (Egypt) [14]	4	2	3	9	Low
Magat & Ozcan 2022 (Turkey) [15]	3	1	2	6	Moderate
Saraç et al. 2024 (Turkey) [16]	4	2	2	8	Low
Lashin et al. 2025 (Egypt) [17]	3	1	2	6	Moderate
Ghergie et al. 2025 (Romania) [18]	3	2	2	7	Moderate
Galic et al. 2025 (South Africa) [19]	4	1	2	7	Moderate
Bravo et al. 2023 (Turkey) [20]	3	1	2	6	Moderate

Discussion

This systematic review synthesized evidence on the comparative accuracy of DA and SA estimation methods versus CA in children and young adults, drawing on multiple recent studies. This review was conducted across diverse populations, including Turkey, Egypt, and others. The findings reveal consistent patterns in the performance of various age estimation techniques and point to both strengths and limitations of DA versus SA methods for different age ranges, sexes, and ethnic backgrounds [23,24].

Summary of Key Findings

The reviewed studies generally show that DA estimation methods (e.g., Cameriere, Demirjian, Willems, Nolla) tend to have high correlations with CA but also exhibit systematic bias (overestimation or underestimation) depending on age group, method, and sex. In a study by Güler et al. (northwestern Turkey), Cameriere's DA slightly underestimated CA in older children and overestimated it in younger ones, whereas SA (via cervical vertebrae) showed significant underestimation in older age groups. The study comparing Demirjian and Willem dental methods with Fishman skeletal assessments showed dental methods more aligned with CA (particularly Willem), while SA (Fishman's) tended to overestimate. In the Egyptian sample, Willems' method had the lowest mean absolute error (<1 year), suggesting superior accuracy in that population when compared with the Cameriere and the Greulich and Pyle methods. These findings, taken collectively, suggest that DA methods are often more precise in certain age ranges (often mid-childhood to early adolescence), while SA methods (hand-wrist, vertebral maturation) may either lag behind in accuracy or show greater underestimation in older children (beyond early adolescence) [25-27].

Methodological Considerations and Sources of Bias

Several methodological factors can influence the accuracy and comparability of DA and SA estimation methods. One major consideration is the age range of participants, as most studies focus on individuals aged 8-16. During adolescence, particularly around the growth spurt, the developmental patterns of teeth and skeletal markers begin to diverge, leading to greater discrepancies in older children. SA methods, in particular, tend to underestimate CA in individuals over 12-13 years, while both dental and skeletal methods generally show better agreement in younger age groups [28-30].

Sex differences also play an important role, as numerous studies have demonstrated that both dental and skeletal maturation occur earlier in females than in males. If the assessment methods do not adequately account for these biological differences or lack proper sex-based calibration, the estimation error increases. Furthermore, some DA estimation techniques, such as Demirjian's method, were developed using reference populations with specific maturation rates that may not correspond to the study population, potentially introducing systematic bias based on sex. Ethnic and population-specific variations further affect the accuracy of these methods, since dental and skeletal development are influenced by genetic, nutritional, and environmental factors. Consequently, techniques derived from one population (e.g., Belgian, Italian, or Canadian) may not be directly applicable to others. For instance, studies on Egyptian samples showed that combining the Willems and Greulich and Pyle methods enhanced accuracy, underscoring the importance of population-specific calibration or the integration of multiple approaches.

Finally, radiographic and measurement-related factors also contribute to variability. The quality of panoramic, cephalometric, and hand-wrist radiographs, along with examiner reliability, can significantly impact the precision of results. Studies with larger, balanced samples and robust methodological designs, such as those from Egyptian and Turkish cohorts, tend to yield more consistent estimates. In contrast, retrospective studies, unequal gender distribution, and small sample sizes are more prone to methodological bias.

Method-Specific Limitations and Comparative Practical Forensic Implications

Each age estimation method presents inherent limitations that influence its precision and applicability across populations and age ranges. The Cameriere method, which relies on the measurement of open apices, is particularly sensitive to the stage of root development. It often overestimates age in younger individuals, where apices remain widely open, and underestimates it in older children, as apical closure progresses. The Demirjian method, one of the most widely used DA estimation techniques, has historically been associated with systematic overestimation in several populations, highlighting its limited adaptability beyond the original reference sample. The Willems method, a modification of Demirjian's approach, generally provides estimates more closely aligned with CA; however, it may also produce discrepancies, particularly in older children or when applied to populations that differ from the calibration cohort. Conversely, SA methods, including Fishman's hand-wrist assessment and CVM, are influenced by variations in growth rate, nutritional status, and overall health, leading to potential inconsistencies across individuals and populations.

Practical Forensic Implications

DA estimation is highly useful in forensic work because tooth development is stable and less influenced by environmental factors, and teeth remain well-preserved in most remains. SA methods can aid assessment in older adolescents but their reliability is more variable due to nutritional and health factors. In cases with fragmentary remains, dental methods often provide more dependable age estimates, and using both approaches together strengthens accuracy near legal age thresholds.

When comparing dental and skeletal methods, evidence suggests that DA assessments tend to outperform skeletal approaches in children aged approximately 9-13 years, particularly when using the Willems or appropriately calibrated Cameriere methods [31,32]. Although some studies, such as that of Güler et al., indicate marginally higher accuracy for skeletal assessments within specific cohorts, the overall trend favors dental methods in this age group. As children progress into late adolescence, skeletal methods, especially those based on hand-wrist or CVM analysis, show a greater tendency to underestimate CA, whereas dental techniques, although not error-free, generally maintain better alignment.

In forensic and clinical contexts where precision is critical, such as determining legal age thresholds, combining dental and skeletal indicators can enhance reliability and minimize bias. The choice between DA and SA approaches should be context-dependent; for instance, in cases where obtaining high-quality dental radiographs is impractical, skeletal assessments may serve as a practical alternative, albeit with reduced accuracy in later developmental stages. Based on the available evidence [33,34], several recommendations can be proposed. First, method selection should be guided by age, sex, and population-specific characteristics, as methods validated in one demographic may not perform equally well in another without local calibration. Second, for children aged 9-13 years, dental methods such as Willems appear to provide the most reliable estimates of CA, while skeletal methods should be used as complementary tools when dental data are limited or uncertain. Third, in older adolescents, clinicians and forensic experts should apply all methods cautiously, recognizing the increased risk of both over- and underestimation. Combining multiple approaches may yield more robust outcomes.

Moreover, studies should adhere to standardized reporting practices, including detailed presentation of error metrics (e.g., mean absolute error, bias), examiner reliability (intra- and inter-observer), and comprehensive demographic data (e.g., sex, ethnicity, and nutritional status) to facilitate cross-study comparisons and reproducibility. Future research should focus on population-specific calibration and on the development of hybrid or machine-learning-based models that integrate dental and skeletal features using large, annotated radiographic datasets. Such advancements may enhance predictive accuracy and promote more reliable, generalizable methods for age estimation across diverse populations.

Limitations of the Review

Despite multiple high-quality studies, heterogeneity in study design (different radiographic techniques, age groupings, and method usage) limits direct comparability. Some studies had a moderate risk of bias related to sample representativeness (e.g., retrospective radiograph collections, unbalanced sex ratios) or a lack of adjustment for confounders such as nutrition and health. Many methods have not been tested in diverse ethnic or socioeconomic settings, so generalizability remains limited.

## Conclusions

In summary, this review confirms that DA estimation methods generally outperform SA methods for children in the early to mid-adolescent years (≈9-13), particularly when methods such as Willems are used and when sufficient sample calibration is available. Skeletal methods may lag in accuracy, especially in older children, though they remain valuable when dental data are limited or in combination. Key to improving accuracy is population‐specific calibration, balanced samples, and, possibly, using combined dental and skeletal approaches. For forensic, clinical, or legal contexts, the choice of method must carefully consider age range, sex, ethnicity, and available imaging.
